# Plasticity of face–hand sensorimotor circuits after a traumatic brachial plexus injury

**DOI:** 10.3389/fnins.2023.1221777

**Published:** 2023-08-07

**Authors:** Fernanda de Figueiredo Torres, Bia Lima Ramalho, Marcelle Ribeiro Rodrigues, Ana Carolina Schmaedeke, Victor Hugo Moraes, Karen T. Reilly, Raquel de Paula Carvalho, Claudia D. Vargas

**Affiliations:** ^1^Laboratory of Neurobiology of Movement, Institute of Biophysics Carlos Chagas Filho, Federal University of Rio de Janeiro, Rio de Janeiro, Brazil; ^2^Laboratory of Neuroscience and Rehabilitation, Institute of Neurology Deolindo Couto, Federal University of Rio de Janeiro, Rio de Janeiro, Brazil; ^3^Research, Innovation and Dissemination Center for Neuromathematics, Institute of Mathematics and Statistics, University of São Paulo, São Paulo, Brazil; ^4^Trajectoires Team, Lyon Neuroscience Research Center, Lyon, France; ^5^University UCBL Lyon 1, University of Lyon, Lyon, France; ^6^Laboratory of Child Development and Motricity, Department of Human Movement Science, Institute of Health and Society, Universidade Federal de São Paulo, Santos, Brazil

**Keywords:** afferent inhibition, corticospinal excitability, transcranial magnetic stimulation, brachial plexus lesion, deafferentation, pain

## Abstract

**Background:**

Interactions between the somatosensory and motor cortices are of fundamental importance for motor control. Although physically distant, face and hand representations are side by side in the sensorimotor cortex and interact functionally. Traumatic brachial plexus injury (TBPI) interferes with upper limb sensorimotor function, causes bilateral cortical reorganization, and is associated with chronic pain. Thus, TBPI may affect sensorimotor interactions between face and hand representations.

**Objective:**

The aim of this study was to investigate changes in hand–hand and face–hand sensorimotor integration in TBPI patients using an afferent inhibition (AI) paradigm.

**Method:**

The experimental design consisted of electrical stimulation (ES) applied to the hand or face followed by transcranial magnetic stimulation (TMS) to the primary motor cortex to activate a hand muscle representation. In the AI paradigm, the motor evoked potential (MEP) in a target muscle is significantly reduced when preceded by an ES at short-latency (SAI) or long-latency (LAI) interstimulus intervals. We tested 18 healthy adults (control group, CG), evaluated on the dominant upper limb, and nine TBPI patients, evaluated on the injured or the uninjured limb. A detailed clinical evaluation complemented the physiological investigation.

**Results:**

Although hand–hand SAI was present in both the CG and the TBPI groups, hand–hand LAI was present in the CG only. Moreover, less AI was observed in TBPI patients than the CG both for face–hand SAI and LAI.

**Conclusion:**

Our results indicate that sensorimotor integration involving both hand and face sensorimotor representations is affected by TBPI.

## 1. Introduction

The classic description of sensorimotor cortex somatotopic organization suggests that motor actions involving different body parts are produced through the combined activation of clearly designated, independent cortical areas in the primary motor cortex (M1) (Jackson, 1870; Gross, 2007). This model has been updated over the years (Donoghue et al., 1992; Park et al., 2001; Sanes and Schieber, 2001; Schieber, 2001; Graziano and Aflalo, 2007). For example, it has been shown that the electrical activation of specific cortical regions in primates generates complex movements, such as reaching, grasping, defending, and hand-to-mouth movements (Graziano, 2006).

Despite being anatomically distant in body space, the face and hand have neighboring cortical representations and interact very closely in many everyday activities, such as self-feeding and communicative manual gestures during speech (Gentilucci and Dalla Volta, 2008; Vainio, 2019). It is not surprising, therefore, that interactions between the sensorimotor functions of the hand and face have been observed in a number of different experimental contexts (Salmelin and Sams, 2002; Tanosaki et al., 2003; Higginbotham et al., 2008; Desmurget et al., 2014). For example, previous research has shown that tactile stimulation of the tip of the index finger can affect touch perception on the face (Muret et al., 2014, 2016). The magnitude of hand and face cortical representations as well as the abundance of sensorimotor interactions between them suggests that there might be anatomical connections between their cortical representations. However, anatomical evidence for this is controversial. Evidence from a histological study in monkeys suggests that there is a clear anatomical separation between hand and face representations in area 3b of the somatosensory cortex in the form of a well-defined myelin septum (Jain et al., 1998). In contrast, studies using marker injections and tracking techniques have identified fibers that project between the sensory and motor representations of the hand and face (Huntley and Jones, 1991; Fang et al., 2002).

The structural and functional consequences of the proximity between hand and face representations are also evident after peripheral lesions (Elbert et al., 1997). For instance, amputation of the hand induces displacement of the cortical representation of the face toward the original hand representation (Ramachandran, 1993; Flor et al., 1995; Pascual-Leone et al., 1996; Weiss et al., 2004; Raffin et al., 2016). This type of plasticity is sometimes accompanied by tactile sensations in the phantom hand when the face is touched (Halligan et al., 1993; Ramachandran, 1993). The stability of cortical representations, the cortical plasticity mechanisms, and their limits, as well as the multiple factors involved in such processes, have all been topics of intense debate (Makin and Bensmaia, 2017; Makin and Flor, 2020).

Given this context, traumatic brachial plexus injury (TBPI) is a highly appropriate model of peripheral lesion for studying cortical reorganization. In most cases, TBPI affects young male adults, is caused mainly by automobile or motorcycle accidents, and can lead to nerve rupture, avulsion at the level of the spinal cord, or significant nerve stretching without rupture (Moran et al., 2005). Factors such as injury mechanism, severity, the presence of pain, concomitant injuries, and the quality of medical and hospital care can influence the extent and heterogeneity of the injury as well as treatment outcomes (Giuffre et al., 2010; Flores, 2011; Franzblau et al., 2015). In addition to the sensorimotor loss in the affected upper limb, the situation is further complicated by the fact that the uninjured upper limb can also undergo significant changes. We recently demonstrated higher tactile detection thresholds on the uninjured upper limb of TBPI patients compared with control participants (Ramalho et al., 2019). Kinematic recordings have also revealed altered motor synergies involving the uninjured limb (Souza et al., 2021; Lustosa et al., 2022).

Numerous studies have shown that TBPI and its surgical reconstruction are associated with plastic modifications in the topographic organization of movement representations in M1 (Narakas, 1984; Mano et al., 1995; Malessy et al., 1998, 2003; Yoshikawa et al., 2012; Qiu et al., 2014; Socolovsky et al., 2017; Torres et al., 2018). Fraiman et al. (2016) showed that cortical changes in TBPI patients are bilateral and mostly specific to the body parts directly affected by the injury. Furthermore, there is evidence that these plastic changes extend beyond the sensorimotor cortices and encompass higher order cognitive networks such as the salience network and the default mode network (DMN) (Lu et al., 2016; Bhat et al., 2017). In line with these higher-order changes, a recent study demonstrated that TBPI patients display altered sensorimotor prediction coding during an action observation and electroencephalography (EEG) paradigm (Rangel et al., 2021). Evidence indicates, therefore, that TBPI is a viable model to better understand neuroplasticity mechanisms at work in the presence of a severe peripheral injury.

In this context, afferent inhibition (AI) protocols provide a simple way to assess sensorimotor integration (Bikmullina et al., 2009; Ferreri et al., 2012; Rossini et al., 2015) and might therefore be a useful technique for investigating the consequences of the sensorimotor reorganization that occurs after TBPI. Tokimura et al. (2000) were the first group to describe a reduction in motor evoked potentials (MEPs) induced by transcranial magnetic stimulation (TMS) when the magnetic pulse was preceded by an electrical stimulus applied to the skin overlying the target muscle. This phenomenon is called short-latency afferent inhibition (SAI) when the interstimulus interval (ISI) is <50 ms and long-latency afferent inhibition (LAI) when longer intervals are used (>100 ms) (Chen et al., 1999). SAI might result from the presence of direct U-shaped connections between homologous representations of the primary somatosensory and motor cortices such as those demonstrated by Catani et al. (2012) in the hand-knob region. Catani et al. (2012) showed three different tracts connecting pre- and post-central regions. The first one connects the hand area of the sensory-motor homunculus, whereas the ventral group corresponds to at least two tracts connecting pre- and post-central regions of the face, mouth, and tongue areas. Two other potential routes between the primary somatosensory cortex (S1) and M1 involving thalamic nuclei may also be involved: signals detected by cutaneous and proprioception receptors being transmitted through a direct thalamic connection to the contralateral M1, or signals arriving first at the contralateral S1, and from there, being transmitted subsequently to M1 (Ruddy et al., 2016). LAI, on the other hand, involves several cortical areas beyond S1, such as the posterior parietal cortex and the secondary somatosensory cortex (S2) (Chen et al., 1999; Turco et al., 2018a). These areas project to M1, where they can mediate MEP amplitudes, but neural pathways between basal nuclei, thalamus, and other cortical areas may also be involved in LAI (Chen et al., 1999; Turco et al., 2018a).

Many SAI and LAI studies have been performed in which electrical stimulation is applied to a skin region close to the target muscle. For example, MEP amplitudes in hand muscles such as the first dorsal interosseous (FDI) or the abductor pollicis brevis (APB) are significantly smaller if the TMS pulse is preceded by electrical stimulation of the median or ulnar nerves or of the skin on the tip of the index finger (Chen et al., 1999; Tokimura et al., 2000; Di Lazzaro et al., 2002, 2005; Sailer et al., 2002; Helmich et al., 2005; Kukaswadia et al., 2005; Tamburin et al., 2005; Bikmullina et al., 2009; Asmussen et al., 2013; Lapole and Tindel, 2014). Although less explored, SAI and LAI have also been reported in face muscles following peripheral electrical stimulation of the trigeminal nerve (face–face SAI) and of the facial nerve (face–face LAI) (Pilurzi et al., 2020). More recently, Ramalho et al. (2022) found that the delivery of a peripheral electrical stimulus to either the skin over the right upper lip or the right cheek inhibited muscular activity in the first dorsal interosseous (face–hand SAI).

In the present study, our aim was to investigate changes in hand–hand and face–hand sensorimotor integration in TBPI patients using the SAI and LAI paradigms and assessing the hemispheres contralateral to the injured and the uninjured limb. If hand–hand AI could be induced in the hand of TBPI patients assessed on the injured side, preserved sensorimotor integration in the spared hand would be demonstrated. If face–hand AI in TBPI patients assessed on the injured side was altered, it could indicate that the plastic changes caused by the TBPI affect the sensorimotor integration between the face and the hand, corroborating the face–hand plastic reorganization observed in upper limb amputees. Finally, as TBPI also causes altered function on the uninjured limb and yields bilateral cortical modifications, AI findings in TBPI patients assessed on the uninjured side should be similar to those assessed on the injured side.

## 2. Materials and methods

### 2.1. Participants

Patients with a diagnosis of unilateral TBPI were recruited using a digital database developed at the Laboratory of Neurosciences and Rehabilitation (LabNeR) (Patroclo et al., 2019) and stored on the Neuroscience Experiment System (NES) platform (Ruiz-Olazar et al., 2022). This database contains clinical and neurophysiological information from adult patients with TBPI treated at the Institute of Neurology Deolindo Couto (INDC) at the Federal University of Rio de Janeiro (UFRJ) or at the National Institute of Traumatology and Orthopedics Jamil Haddad (INTO). Recruitment took place between September 2019 and March 2020 and a selection based on clinical data (no history of severe traumatic brain injury or prolonged loss of consciousness, no long-term use of drugs affecting the central nervous system, and absence of metal implants) produced a preliminary list of 23 patients; nine TBPI patients who met the inclusion and exclusion criteria agreed to participate in the study. Although the number of patients was small, it was similar to that found in previous TMS studies with patients (Tokimura et al., 2000; Mercier et al., 2006; Batista e Sá et al., 2015). We also recruited 18 healthy volunteers from the UFRJ community (students and employees) to form a control group (CG).

Inclusion criteria for all participants included the following: age between 18 and 55 years; any gender; preserved communication ability; tolerance to remain seated for at least 2 h. Exclusion criteria were a history of psychiatric illness, including substance abuse, or cognitive impairment; history of diseases and/or sequelae of the central or peripheral nervous system; history of chronic pain before the TBPI (for the TBPI group) or any report of chronic pain (for the control group); and answering YES to any question in the safety screening questionnaire for TMS application (adapted from Rossi et al., 2011). All participants took part in the experimental protocol at LabNeR between May 2019 and January 2022. There was, however, a recess of all experimental activities between March 2020 and June 2021, while COVID-19 lockdowns were in place. Additionally, clinical assessments of TBPI patients were conducted between January 2019 and March 2020, and some complementary information was obtained by telephone between March and May 2020. The TMS protocol and the TBPI database enrollment were approved by the research ethics committee of INDC-UFRJ (registered numbers: 2.411.426 and 2.087.610, respectively), and all participants gave written informed consent.

**Figure 1:**
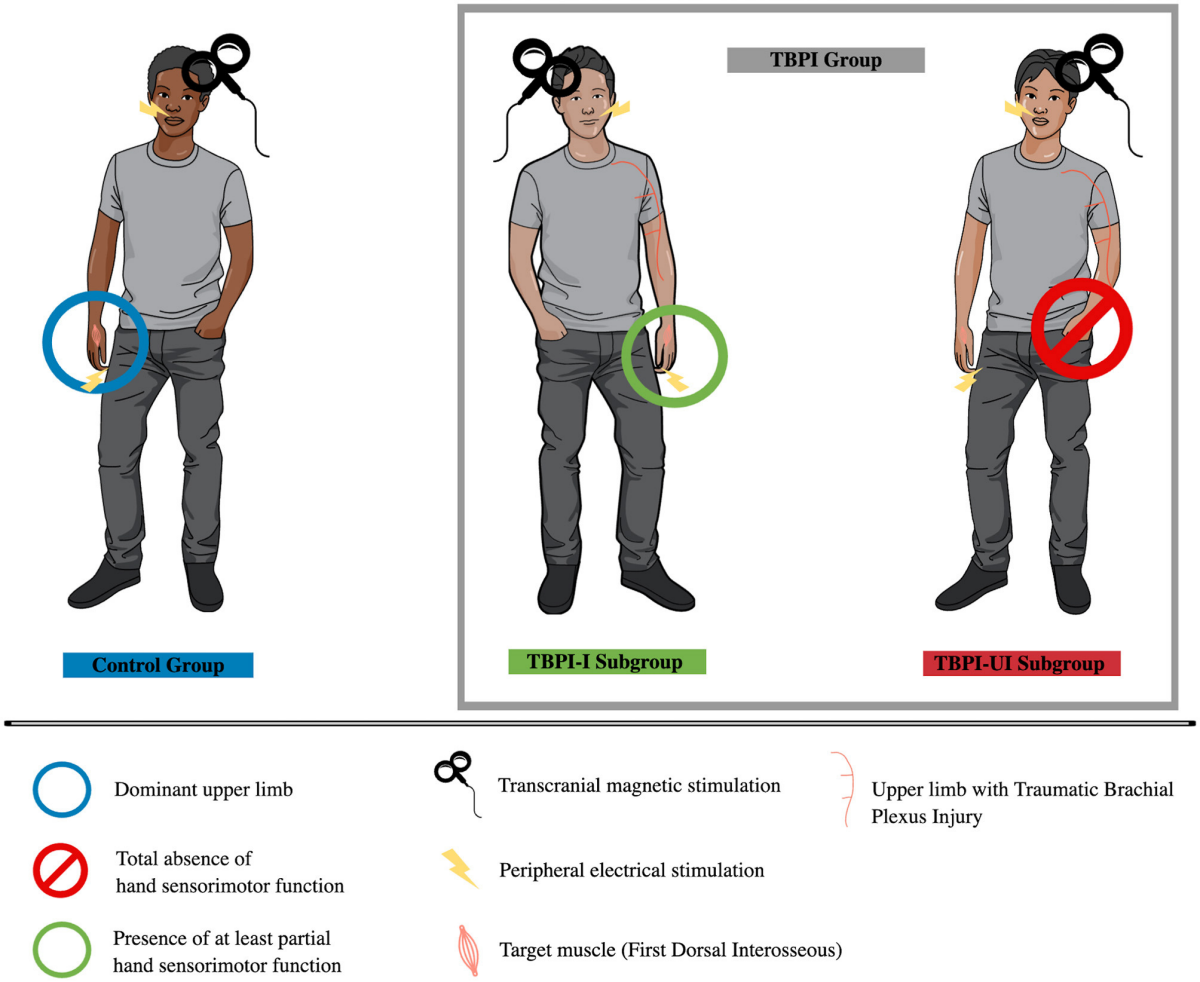
Experimental groups. Control group (in blue, on the left) was assessed on the dominant upper limb. The TBPI group was composed of TBPI-I and TBPI-UI subgroups. The TBPI-I subgroup (in green, center) was formed by participants with at least partial sensorimotor function in the hand of the injured side, allowing it to be evaluated using the afferent inhibition protocol. The TBPI-UI subgroup (in red, on the right) was formed by participants with a total absence of sensorimotor function in the affected hand. Thus, they were assessed on the uninjured side. Peripheral electrical stimulation (indicated by a ray symbol) was applied either on the tip of the ipsilateral index finger or above the upper lip, depending on the experimental condition. Transcranial magnetic stimulation was applied over the scalp contralateral to the assessed limb.

Given that TBPI can lead to sensorimotor alterations on the uninjured side (Ramalho et al., 2019; Souza et al., 2021; Lustosa et al., 2022) and bilateral injury-induced cortical modifications (Hsieh et al., 2002; Liu et al., 2013; Fraiman et al., 2016; Rangel et al., 2021), we reasoned that investigating AI in the uninjured limb of those patients with complete or almost complete TBPI could provide valuable information about cortical reorganization. Therefore, the group of TBPI participants was subdivided based on their clinical characteristics and diagnosis. Those with at least partial sensorimotor function in the hand (i.e., incomplete injury with an upper trunk or extended upper trunk injury diagnosis) were allocated to the *TBPI assessed on the injured side* (TBPI-I) subgroup. Those without any hand sensorimotor function in the injured limb (i.e., diagnosis of almost complete injury) were allocated to the *TBPI assessed on the uninjured side* (TBPI-UI) subgroup. Thus, the final research paradigm was composed of the TBPI group (including TBPI-I, TBPI-UI subgroups) and the control group (Figure 1).

### 2.2. Clinical assessment and pain evaluation

All TBPI participants were clinically evaluated using an assessment protocol developed by the LabNeR research group (Patroclo et al., 2019). Collected data included demographic data, injury details, physical examination data (strength and tactile sensitivity), and surgical history. Upper limb functionality was also assessed using the Disabilities Arm, Shoulder, and Hand (DASH) questionnaire (Orfale et al., 2005). A visual analog scale (VAS) was used to determine pain intensity at the time of assessment (Downie et al., 1978). Complete clinical assessment time was approximately 2 h. Additionally, on the day of the experimental protocol, all participants completed the safety screening questionnaire for the application of TMS (adapted from Rossi et al., 2011) and the Edinburgh Handedness Inventory (Oldfield, 1971). For the latter, TBPI participants responded considering their hand use before the accident.

### 2.3. Transcranial magnetic stimulation and electromyography

TMS was applied on the scalp contralateral to the studied limb: the dominant side for the control group, the uninjured side for the TPBI-UI subgroup, and the injured side for the TPBI-I subgroup. TMS was performed with a Magstim 200^2^ stimulator (The Magstim Company, Carmarthenshire, UK) using a figure-of-eight coil with a 70 mm internal diameter for each wing. The coil was positioned tangentially to the skull over M1, with the handle pointing backward at an angle of 45° from the midline. A neuronavigation system (InVesalius Navigator 3.1.1−3 Space TM Fastrack^®^–Polhemus Isotrack II) was used to ensure consistent TMS coil positioning throughout the experimental session (Souza et al., 2018).

Motor output was recorded with surface electromyography (EMG) electrodes positioned over the muscle belly of the FDI muscle. Adhesive Ag/AgCl surface electrodes (Neuroline 715, Ambu, Copenhagen, Denmark) were positioned in a bipolar configuration with 20 mm between electrodes. The ground electrode was positioned on the styloid process of the ulna ipsilateral to the recording site. The EMG signal was sampled at 2 kHz with a gain of 1,000, filtered through a two-pole Butterworth bandpass filter with a frequency range of 20–500 Hz, and digitized using a CED 1902 amplifier and a CED Power 1401 data acquisition unit (Cambridge Electronic Design Limited, Cambridge UK). The EMG signal was then processed with Signal software version 6.05 (Cambridge Electronic Design Limited, Cambridge UK) and stored on a computer for further data analysis.

During the experimental sessions, participants were seated in a comfortable chair with an arm support that maintained the shoulder at a neutral position, elbow flexed at approximately 90°, forearm, and wrist in a neutral position and hand relaxed. Participants were instructed to keep the upper limb relaxed, both feet flat on the floor, avoid talking during the experiment, and remain awake with their eyes open (Supplementary Figure S1 illustrates participants' positioning during the experimental session). The EMG signal was visually monitored throughout the experiment to ensure that the target muscle was completely relaxed. If muscle activity was detected, the participant was verbally instructed to relax.

The following TMS measurements were obtained:

(a) FDI hot spot: the coil was initially positioned over C3 or C4, and the FDI hotspot was found by stimulating various sites at a slightly suprathreshold intensity in order to identify the site that generated stable MEPs with the largest peak-to-peak amplitudes, reproducible on at least five consecutively applied stimuli.

(b) Resting motor threshold (rMT): once the FDI hotspot had been identified, we measured the minimum stimulation intensity required to obtain MEPs with amplitude of at least 50 μV (peak-to-peak) on at least 5 of 10 consecutively applied stimuli (Groppa et al., 2012).

(c) Motor threshold to elicit 1 mV responses (1 mV/MT): with the coil positioned over the hotspot, we gradually increased the stimulation intensity in order to obtain MEPs with amplitude of at least 1 mV (peak to peak) on at least 5 of 10 consecutively applied stimuli. One mV/MT intensity was used for all AI experimental conditions (Turco et al., 2018a; Ramalho et al., 2022).

### 2.4. Peripheral electrical stimulation

For the afferent inhibition protocol, a single electrocutaneous stimulus was delivered via a constant current stimulator (square wave, 200 μs, STMISOLA, BIOPAC Systems Inc., USA) using adhesive electrodes (Neuroline 715, Ambu, Copenhagen, Denmark), placed in a bipolar configuration. They were placed above the upper lip (next to the lip philtrum, placed side by side with a 1 cm separation between the center of each electrode) or on the first and second phalanges on the palmar surface of the index finger.

The peripheral electrical threshold (pET) was obtained for the finger and the face by measuring the lowest, perceptible stimulation intensity that did not evoke any report of pain. Starting at 1.0 mA, the intensity was increased in 0.5 mA steps until the participant was able to feel the stimulation. Then, the intensity was decreased in 0.1 steps until the participant could not feel the stimulation anymore. The intensity was then once again increased in 0.1 steps until the participant was able to identify 10 consecutive pulses. The pET was then used to determine the peripheral electrical stimulation intensity (pESI), which was set at 3 x pET for the finger and 2 x pET for the face.

### 2.5. Afferent inhibition protocol

A single electrocutaneous stimulation was applied to the skin above the upper lip or on the index fingertip and was followed by a single TMS pulse at 1 mV/MT intensity over the FDI hotspot. In order to investigate both SAI and LAI, a range of interstimulus intervals (ISIs) were examined: 15, 25, 35, 45, 55, and 65 ms (SAI) and 100, 200, 300, and 400 ms (LAI). Single TMS pulses that were not preceded by an electrical stimulus were used as a control (TMS-only). The choice of ISIs was based on previous literature showing significant SAI in FDI when electrocutaneous stimulation is applied to the index fingertip at ISIs between 25 and 50 ms (Helmich et al., 2005; Tamburin et al., 2005; Bikmullina et al., 2009) and when it is applied to the face at ISIs between 45 and 65 ms (Ramalho et al., 2022). Similarly, LAI in the FDI has been reported at ISIs between 180 and 200 ms when electrocutaneous stimulation is applied to the index finger (Chen et al., 1999; Voller et al., 2006; Turco et al., 2018a) and between 100 and 600 ms after median nerve or middle finger stimulation (Sailer et al., 2003). LAI has not yet been explored for face–hand interactions, so LAI ISIs were chosen to cover a wide range of possibilities (100–400 ms) for a putative observation of an inhibition effect.

Each testing session consisted of four experimental conditions based on the sensory stimulation site (upper lip or index finger) and the tested ISIs (SAI or LAI). Each condition was tested in a separate block and the order of the conditions was randomized for all participants. Within a given block, 14 trials of paired electrocutaneous and TMS stimuli for each ISI plus 14 TMS-only trials were randomly applied. Intertrial intervals varied between 2.5 and 4 s. The two SAI blocks consisted of 98 trials (6 ISIs x 14 plus 14 TMS-only) and the LAI blocks consisted of 70 trials (4 ISIs x 14 plus 14 TMS-only). For each condition, there was a brief pause of approximately 1 min after every 49 pulses (SAI) or 35 pulses (LAI). The average duration of the entire experimental session was around 2.5 h.

### 2.6. Data processing and statistical analysis

**Figure 2:**
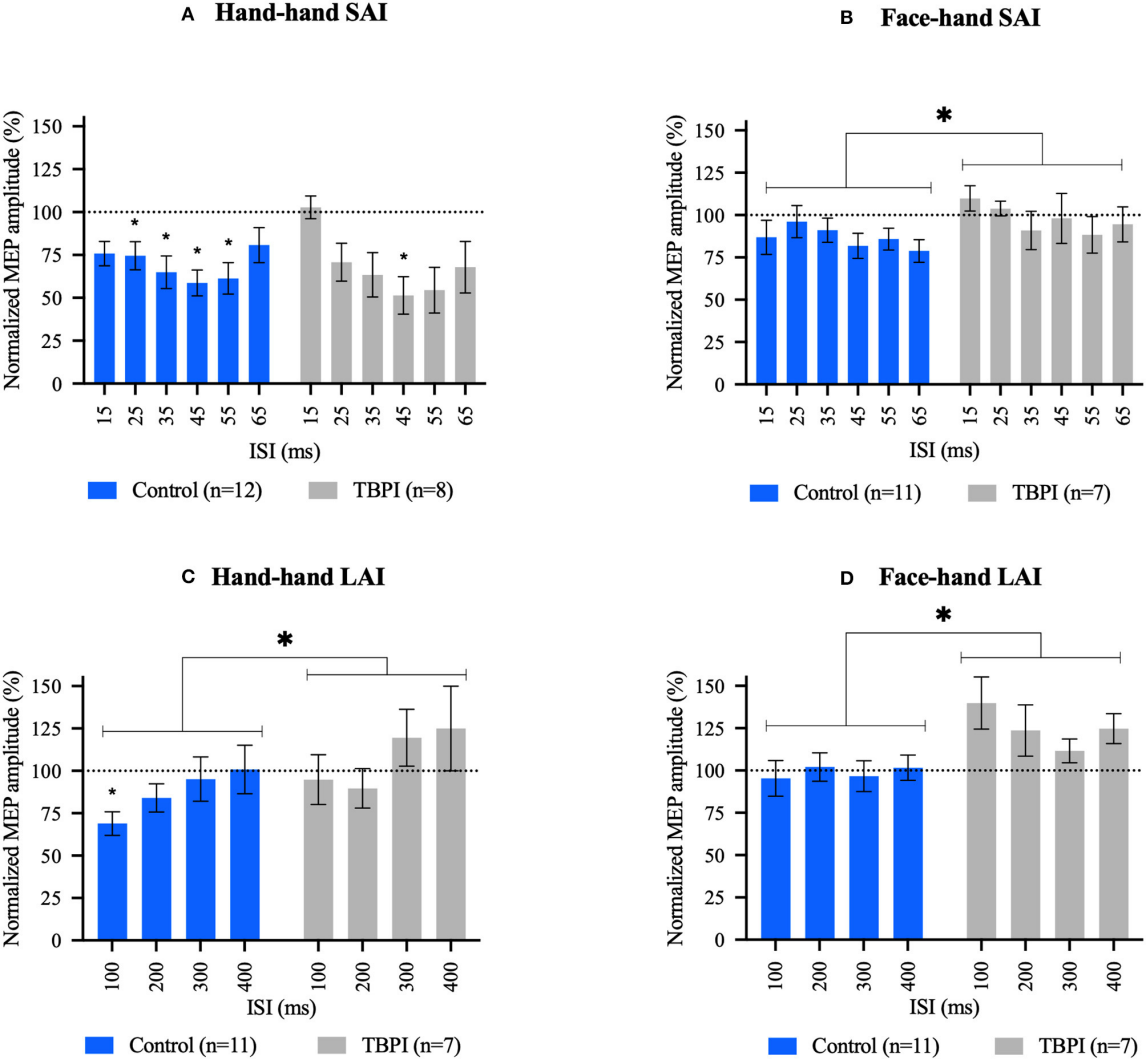
Short afferent inhibition (SAI) and long afferent inhibition (LAI) results. **(A, C)** Hand–hand interaction: peripheral electrical stimulation applied on the tip of the index finger followed by contralateral transcranial magnetic stimulation over the first dorsal interosseous hot spot. **(B, D)** Face–hand interaction: peripheral electrical stimulation applied on the face, above the upper lip, followed by contralateral transcranial magnetic stimulation over the first dorsal interosseous hot spot. Different interstimulus intervals were applied (15–65 ms for SAI and 100–400 ms for LAI). Group mean motor evoked potential amplitudes for each interstimulus interval were normalized to the TMS-only mean MEP amplitude (transcranial magnetic stimulation without previous peripheral electrical stimulation) in that experimental condition. Control group (blue); TBPI group, all TBPI participants (gray). Bars represent the standard error of the mean. Values below the dotted line at 100% indicate an inhibition effect, and values above the dotted line indicate a disinhibition effect. Repeated-measures one-way ANOVA and Dunnett's post-test were performed for within-group analysis. Two-way ANOVAs and Šidák's post-test were used for between-group comparisons. *p < 0.05; *above bar indicates significant results at within-group analysis; and *above brackets indicate significant results in between-group analysis. Repeated-measures one-way ANOVA and Dunnett's post-test results. **(A)** CG: F_3.231, 35.54_ = 5.824, *p* = 0.002; 25 ms: *p* = 0.036, 95% C.I. = [0.014; 0.432], 35 ms: *p* = 0.012, 95% C.I. = [0.067; 0.539], 45 ms: *p* = 0.005, 95% C.I. = [0.108; 0.577], and 55 ms: *p* = 0.009, 95% C.I. = [0.086; 0.597]. TBPI: F_2.615, 18.30_ = 5.753, *p* = 0.008; 45 ms: *p* = 0.028, 95% C.I. = [0.050; 0.773]. **(B)** CG: F_2.735, 27.35_ = 1.511, *p* = 0.236. TBPI: F_2.112, 12.67_ = 1.094, *p* = 0.368. **(C)** CG: F_2.015, 20.15_ = 3.772, *p* = 0.040; 100 ms: *p* = 0.008, 95% C.I. = [0.088; 0.540]. TBPI: F_2.492, 14.95_ = 2.780, *p* = 0.085. **(D)** CG: F_2.139, 21.39_ = 0.6515, *p* = 0.541. TBPI: F_1.855, 11.13_ = 2.159, *p* = 0.163. Two-way ANOVAs results can be found in the text.

Peak-to-peak MEP amplitudes (mV) were measured offline using custom-written Signal scripts (Cambridge Electronics Design, Cambridge, UK). Based on visual inspection, trials with clear evidence of muscle contraction or with EMG artifacts that prevented MEP measurement were excluded. After measuring MEP amplitudes, trials were excluded if their amplitudes were above or below 1.5 times the lower or upper limit of the interquartile range for that condition for that participant. If more than 29 trials were excluded for an SAI condition or 21 trials for an LAI condition (>30% of the trials), the participant's data for that condition were excluded from the final analysis. A total of three datasets (for hand LAI, face SAI, and face LAI) from one TBPI patient were excluded according to this criterion. Additionally, data from conditions in which the TMS-only mean MEP amplitude were of <0.3 mV were also excluded. If the mean MEP amplitude for the TMS-only condition was too small, conditioned MEPs would be even smaller, making it difficult to differentiate a highly inhibited MEP from the absence of MEP (Turco et al., 2018a). Based on this criterion, a total of 18 datasets from various conditions (four for hand SAI, seven for hand LAI, three for face SAI and four for face LAI) from eight different participants (seven control participants and one TBPI patient) were excluded for this reason. Figure 2 shows the final numbers of participants analyzed for each experimental condition. Please refer to the “Data Availability Statement” section to request detailed data containing raw MEP peak-to-peak amplitudes for all trials and all experimental conditions for each participant.

Mean MEP peak-to-peak amplitudes were obtained for each participant for each of the four experimental conditions. Mean MEP amplitudes for a given ISI were normalized to the mean TMS-only MEP amplitude measured in that experimental condition. Raw and normalized data were then averaged across participants within a given group (control group, TBPI group, and TBPI-I/TBPI-UI subgroups). For normalized data, values below 100% indicate inhibition and values above 100% indicate disinhibition. After the exclusion process described above, the Shapiro–Wilk test was used to verify whether preprocessed data could be described by a normal distribution. As this was the case, we used parametric statistics to further analyze the data.

To compare experimental parameters (TMS-only mean MEP amplitude, 1 mV/MT, finger, and face pET and pESI), unpaired *t*-tests with Welch's correction were performed. We first compared the two TBPI subgroups to check whether they could be analyzed as a single group (TBPI group). As this was the case, we then compared the TBPI group and the control group.

Two separate one-way repeated-measures ANOVAs with Dunnett's multiple comparisons post-tests were performed within the TBPI and control group data to compare mean MEP amplitudes in each ISI with the mean TMS-only MEP amplitude. This analysis allowed us to investigate which ISIs have significantly smaller MEPs than the TMS-only condition (i.e., ISIs at which AI was present).

To compare MEP amplitudes between the subgroups, we first performed two-way repeated-measures ANOVA and Šidák's multiple comparisons tests using the non-normalized MEP amplitudes for each of the four experimental conditions to compare TBPI-I and TBPI-UI subgroups and check whether they could be assembled in a single TBPI group. For that, we used ISI and subgroups (TBPI-I x TBPI-UI) as factors. Next, group comparisons between the control group and the TBPI group were performed using normalized data (%TMS-only MEP amplitude) and two-way ANOVAs with ISI and group (CG x TBPI group) as factors. Šidák's multiple comparisons tests were used in the *post-hoc* analysis, when appropriate. Alternatively, we performed a two-way ANOVA and Tukey's multiple comparisons test for between-group comparisons considering TBPI-I and TBPI-UI subgroups as independent groups using ISI and group (CG x TBPI-I x TBPI-UI) as factors (Supplementary material). The alpha level was set at 5%. All data were analyzed with Prism 9 software (GraphPad Software, Inc., California, USA).

## 3. Results

### 3.1. Sociodemographic data, clinical assessment, and pain evaluation

In total, 18 control participants (15 male, 3 female) and 9 TBPI patients (all male) were enrolled in this study. The mean age for the control group was 30 ± 10 (20–54) and for the TBPI group 36 ± 11 (24–55) years; 16 participants in the control group and 8 participants in the TBPI group were right-handed, according to the Edinburgh Handedness Inventory results. In total, five TBPI patients were evaluated on the injured side (TBPI-I subgroup) and four on the uninjured side (TBPI-UI subgroup). TBPI was caused by motorcycle accidents for all participants. Data on TBPI history, diagnosis, previous treatments, physical examination, upper limb functionality, and pain intensity are shown in Table 1.

**Table 1:** Clinical characteristics of TBPI participants.

	**TBPI-I1**	**TBPI-I2**	**TBPI-I3**	**TBPI-I4**	**TBPI-I5**	**TBPI-UI-1**	**TBPI-UI2**	**TBPI-UI3**	**TBPI-UI4**
Handedness	R	R	R	R	R	R	R	L	R
Injured arm	L	L	R	R	R	L	L	L	L
Tested brain hemisphere	R	R	L	L	L	L	L	L	L
Age at testing	36	30	45	47	25	24	29	28	54
Diagnosis	Extended upper trunk	Extended upper trunk	Upper trunk	Extended upper trunk	Extended upper trunk	Complete	Complete	Upper trunk	Complete
Time between surgery and TMS protocol	4 y 10 m	5 y 10 m	1 y 7 m	5 y 2 m	4 y 6 m	1 y 5 m	12 y 1 m	4 y 1 m	3 y 5 m
Surgery	BP Exploration	Oberlin + A/SS	Oberlin + A/SS + T/A	Oberlin	Oberlin	Neurotization S/P/A	-	P/MC	A/MC
Time between injury and surgery	10 m 21 d	5 m 5 d	3 m 3 d	11 m 19 d	1 y 1 m 20 d	7 m 12 d	-	4 m 23 d	6 m 24 d
Orthopedic surgeries	clavicle, arm	clavicle	leg	arm, forearm, thigh, knee	-	clavicle	-	arm, hand	cervical vertebra
Physical therapy	N	N	Y	Y	N	Y	N	Y	Y
Tactile sensitivity	↑C5, T3; ↓C6-8	↓C3, C5, C7-8; ØC4, C6	ØC4-T2	↑C4-8; ↓T1-3	↑C4; ↓C5	↓C5-6, T1; ØC6-8	ØC5-T1	↑C5; ↓C6-T1	↓C3-5; ØC6-8
Pain sensitivity	↑C3-4, T1, T3; ↓T2; ØC5-8	↓C8; ØC4-7	ØC6-8	↑C4; ↓C5, C8-T1; ØC6-7, T2-3	↓C4; ØC5	↓C4; ØC5-T1	ØC5-T1	↓C6-8; ØC5, T1	↓C4-5, T1-2; ØC6-8
Strength	↓shoulder, elbow	↓shoulder	↓shoulder	↓shoulder, elbow, hand	↓shoulder, elbow	Ø shoulder, elbow, hand	Ø shoulder, elbow, hand	↓shoulder, elbow, Ø hand	Ø shoulder, elbow, hand
DASH	33.33	25	40.52	31.67	40.83	19.64	22.5	49.17	68.33
VAS	3	8	2	6	10	-	10	10	7

R, right; L, left; y, years; m, months; d, days; N, no; Y, yes; BP exploration, brachial plexus, exploration; Oberlin, transfer of an ulnar fascicle to the biceps branch of the musculocutaneous nerve; A/SS, transfer of the accessory nerve to the suprascapular nerve; T/A, axillary nerve neurotization by a triceps motor branch; neurotization S/P/A, neurotization with sural, phrenic, and accessory nerve grafts; P/MC, phrenic nerve transfer to the musculocutaneous nerve; A/MC, accessory nerve transfer to the musculocutaneous nerve; ↑, increased; ↓, decreased; Ø, absent; C3, C4, C5, C6, C7, C8, T1, T2: matching dermatomes; DASH, Disabilities Arm, Shoulder, and Hand questionnaire score, ranging from 0 (maximum functionality) to 100 (maximum disability); VAS, visual analog scale, assessment of pain intensity at the moment of evaluation, ranging from 0 (no pain) to 10 (maximum pain intensity).

### 3.2. Transcranial magnetic stimulation and electrocutaneous stimulation parameters

Transcranial magnetic stimulation and peripheral electrical stimulation parameters (mean ± standard deviation) for each group are shown in Table 2. Experimental parameters for the two TBPI subgroups did not differ (TMS-only mean MEP amplitude: *t* = 0.2537, df = 3.751, *p* = 0.813; 1 mV/MT: *t* = 0.7369, df = 6.998, *p* = 0.485; finger pET: *t* = 0.8690, df = 4.942, *p* = 0.425; face pET: *t* = 1.538, df = 4.594, *p* = 0.190; finger pESI: *t* = 1.423, df = 5.157, *p* = 0.212; face pESI: *t* = 1.583, df = 4.667, *p* = 0.179). Supplementary Table S1 displays TMS and peripheral electrical stimulation parameters (mean ± standard deviation) for the TBPI subgroups (TBPI-I and TBPI-UI).

**Table 2:** Transcranial magnetic stimulation and peripheral electrical stimulation parameters.

	**Control group (n=18)**	**TBPI group (n=9)**
TMS-only MEP amplitude (mV)	0.71 ± 0.42	0.84 ± 0.40
1 mV/MT (% MSO)	52.17 ± 12.17	52.33 ± 7.91
Finger pET (mA)	2.69 ± 0.82^*^	4.30 ± 1.34^*^
Face pET (mA)	2.02 ± 0.99	2.30 ± 1.70
Finger pESI (mA)	7.96 ± 2.37^*^	12.27 ± 4.31^*^
Face pESI (mA)	4.46 ± 2.10	4.56 ± 3.43

Results are mean ± standard deviations. Between-group comparisons were performed using unpaired t-tests with Welch's correction. TBPI, traumatic brachial plexus injury participants; TMS-only MEP amplitude, mean motor evoked potential amplitude when transcranial magnetic stimulation was applied without previous peripheral electrical stimulation; 1 mV/MT, motor threshold to elicit 1-mV responses, MSO, maximum stimulator output; pET, peripheral electrical threshold for the finger and the face; pESI, peripheral electrical stimulation intensity used during AI experiments set at 3xpET for the finger and 2xpET for the face. ^*^significant difference between the TBPI group and the control group.

There were also no significant differences between the control and the TBPI groups for TMS-only mean MEP amplitude (*t* = 0.4080, df = 14.73, *p* = 0.689) nor for the amplitude of the 1mV/MT (*t* = 0.04279, df = 22.99, *p* = 0.966). Similarly, face pET (*t* = 0.4464, df = 11.53, *p* = 0.664) and face pESI (*t* = 0.07735, df = 11.92, *p* = 0.940) were similar across the two groups. Finally, the finger pET (*t* = 3.308, df = 11.25, *p* = 0.007) and pESI (*t* = 2.783, df = 10.63, *p* = 0.018) were significantly higher in the TBPI group than in the control group.

### 3.3. Afferent inhibition

To compare TBPI-I and TBPI-UI subgroups and check whether they could be analyzed as a single group, we performed a two-way repeated-measures ANOVA with Šidák's multiple comparisons tests using the non-normalized MEP amplitudes for each of the four experimental conditions. As none of the ANOVAs revealed an ISI x Group interaction (hand–hand SAI: F_6, 36_ = 1.035, *p* = 0.419; face–hand SAI: F_6, 30_ = 0.6850, *p* = 0.663; hand–hand LAI: F_4, 20_ = 1.102, *p* = 0.383; face–hand LAI: F_4, 20_ = 1.893, *p* = 0.151) or a main effect of Group (hand–hand SAI: F_1, 6_ = 0.5295, *p* = 0.494; face–hand SAI: F_1, 5_ = 0.5038, *p* = 0.510; hand–hand LAI: F_1, 5_ = 0.02721, *p* = 0.875; face–hand LAI: F_1, 5_, = 1.321, *p* = 0.302), all TBPI patients were pooled and analyzed as a single group (TBPI group). Figure 2 shows the normalized mean MEP amplitudes (means ± SE) for the control group and TBPI participants at each ISI for SAI and LAI intervals when peripheral electrical stimulation was applied to the index fingertip (Figures 2A, C) or the upper lip (Figures 2B, D).

Alternatively, we have performed a two-way ANOVA comparing the three groups (CG x TBPI-I x TBPI-UI) and using ISI and groups as factors and Tukey's multiple comparisons test as *post-hoc*. These results are available in the Supplementary material (Supplementary Figure S2). It should be noted that results remained roughly similar in this context. Supplementary Figure S2 shows the normalized mean MEP amplitudes (means ± SE) for the control group and TBPI subgroups (TBPI-I in green bars and TBPI-UI in red bars) at each ISI for SAI and LAI intervals when peripheral electrical stimulation was applied to the index fingertip (Supplementary Figures S2A, C) or the upper lip (Supplementary Figures S2B, D). Supplementary Figure S3 shows individual results (normalized mean MEP amplitudes at each ISI) for each experimental group at the four experimental conditions. A complete dataset containing MEP amplitudes for all trials for each participant is available upon request (refer to the “Data Availability Statement” section).

### 3.4. Short afferent inhibition in hand–hand and face–hand sensorimotor circuits

For the hand–hand SAI condition (Figure 2A), average MEP amplitudes were below TMS-only MEP amplitude (100%) at all ISIs for the control group (blue bars). Inhibition was maximal at 45 ms (41.28%) and significantly different from the TMS-only condition at all ISIs except 15 and 65 ms. The pattern of responses in the TBPI participants group (gray bars) was similar to that observed in the control group, although inhibition was only significant at the 45 ms ISI, reaching 48.63%. To examine differences between the control group and TBPI patients, a two-way ANOVA was performed to compare normalized mean MEP amplitudes at each ISI between the two groups. This revealed no ISI x Group interaction (F_5,108_ = 0.9670, *p* = 0.441), no main effect of Group (CG x TBPI: F_1,108_ = 0.02387, *p* = 0.878), but a significant effect of ISI (F_5,108_ = 3.061, *p* = 0.0127).

For the face–hand SAI condition (Figure 2B), average MEP amplitudes were below TMS-only MEP amplitude (100%) at all ISIs for the control group (blue bars). Inhibition was maximal at 65 ms (21.19%). The TBPI group displayed a small amount of inhibition at 35, 55, and 65 ms, with a maximal effect at 55 ms (11.67% of inhibition). One-way repeated-measures ANOVAs within each group revealed no significant inhibition. To examine differences between the control group and TBPI patients, a two-way ANOVA was performed. This revealed no ISI x Group interaction (F_5,96_ = 0.4728, *p* = 0.796), no main effect of ISI (F_5,96_ = 0.7679, *p* = 0.575), but a significant main effect of Group (F_1,96_ = 4.179, *p* = 0.044), with average normalized MEP amplitudes being higher for TBPI patients than for the control group.

### 3.5. Long afferent inhibition in hand–hand and face–hand sensorimotor circuits

For the hand–hand LAI condition (Figure 2C), the control group had mean MEP amplitudes below 100% at 100, 200, and 300 ms, although inhibition was significant only at 100 ms, reaching 31.10%. The TBPI group displayed average MEP amplitudes slightly below 100% at 100 and 200 ms (maximum inhibition of 10.30% at 200 ms). In contrast, the mean MEP amplitudes for the TBPI group were above 100% at both 300 and 400 ms (24.90%). The repeated-measures one-way ANOVA revealed no significant effect of ISI for the TBPI group. The two-way ANOVA comparing the two groups (CG x TBPI) revealed a main effect of Group (F_1,64_ = 4.112, *p* = 0.047), once again with average normalized MEP amplitudes across all ISIs higher for TBPI patients than for the control group. There was no significant Group x ISI interaction (F_3,64_ = 0.2358, *p* = 0.871) nor a main effect of ISI (F_3,64_ = 2.360, *p* = 0.080).

Finally, for the face–hand LAI condition (Figure 2D), mean MEP amplitudes were close to 100% for all four ISIs for the control group, while in the TBPI group, all values were above 100% (maximum of 39.90% at 100 ms). One-way repeated-measures ANOVAs revealed no effect of ISI in either group. The two-way ANOVA comparing the two groups revealed a main effect of Group (F_1,64_ = 12.34, *p* = 0.0008), but not of ISI (F_3,64_ = 0.5795, *p* = 0.631), and the ISI x Group interaction was not significant (F_3,64_ = 0.7464, *p* = 0.528). Once again, TBPI patients displayed significantly higher average normalized MEP amplitudes across all ISI than the control group.

## 4. Discussion

This is the first study to investigate patterns of hand–hand and face–hand sensorimotor integration in TBPI patients. Data from the SAI condition reveal that hand–hand sensorimotor integration is at least partially preserved in TBPI patients. However, for all the other conditions (face–hand SAI; hand–hand LAI; face–hand LAI), average MEP amplitudes were higher in TPBI patients than in control participants.

### 4.1. Hand–hand and face–hand SAI in control participants and TBPI participants

In line with previous studies of SAI in FDI, we found significant inhibition in the control group between 25 and 55 ms (Tokimura et al., 2000; Tamburin et al., 2002, 2005; Bikmullina et al., 2009; Asmussen et al., 2013). A similar pattern was observed in the TBPI group, who showed significant inhibition at 45 ms. The absence of a significant effect of group in the two-way ANOVA suggests that the pattern of hand–hand inhibition was similar in both control and TBPI participants.

SAI protocols have already been widely used to examine sensorimotor interactions within the same body part (Chen et al., 1999; Tokimura et al., 2000; Tamburin et al., 2001; Di Lazzaro et al., 2002, 2005; Sailer et al., 2002, 2003; Helmich et al., 2005; Kukaswadia et al., 2005; Bikmullina et al., 2009; Asmussen et al., 2013, 2014; Pilurzi et al., 2013; Lapole and Tindel, 2014), especially when the electrical stimulus is close to the target muscle (Dubbioso et al., 2017). In fact, Dubbioso et al. (2017) found facilitation when stimulating a finger distant to the target muscle (heterotopic stimulation), contrasting with the inhibition effect observed during homotopic stimulation (finger close to the muscle targeted by TMS), revealing a somatotopic expression of SAI. However, previous studies (Classen et al., 2000; Tamburin et al., 2001) did find inhibition during heterotopic stimulation, although lesser than when the stimulation was homotopic. A previous study from our group explored sensorimotor interactions between distant body parts. Significant face–hand SAI was observed at 45, 55, and 65 ms in a large sample of healthy participants (Ramalho et al., 2022). In the present study, visual inspection of the data clearly shows some MEP inhibition in the control group in the face–hand condition (average normalized MEP amplitudes across the six ISIs were 86.45%). Although this was not significant, there was a significant difference between the two groups, indicating that the TBPI group had significantly higher overall amplitudes than the control group. Given that the 95% confidence intervals all include zero, this result suggests the absence of hand–face inhibition in the TBPI group, maybe reflecting reduced coupling of the face and hand representations after TBPI.

SAI has been used as a tool to investigate different pathologies. For neurological disorders such as multiple sclerosis, stroke, and spinal cord injury, hand SAI is reduced in patients when compared to control participants (Vucic et al., 2023). A large number of studies have also demonstrated decreased SAI in Alzheimer's disease (Di Lazzaro et al., 2002, 2004; Nardone et al., 2008). SAI has also been investigated as a potential biomarker to guide treatment in mild cognitive impairment and dementia (Di Lazzaro et al., 2021). Regarding Parkinson's disease, SAI studies have shown variable results, from reduced to normal or even enhanced SAI, depending on disease severity and duration, therapy, and cognitive status. Its validation as a biomarker of central cholinergic activity is under research (Dubbioso et al., 2019). However, evidence for SAI changes after a peripheral injury such as TBPI has been lacking in literature outside of the chronic pain context. TBPI patients likely have altered nerve conduction velocity due to the injury (Ferrante and Wilbourn, 2002), which could explain why, despite the absence of an effect of group, TBPI patients displayed hand–hand SAI only at the 45 ms ISI.

Another important aspect that must be addressed is that prior study has demonstrated that arm dominance has an impact on movement control and sensory feedback (Mutha et al., 2012). Furthermore, Helmich et al. (2005) found that SAI elicited in the right FDI in healthy right-handed individuals was stronger than that elicited in the left hand. In the present study, however, the number of parameters (handedness X injured arm x assessed brain hemisphere) that could affect a handedness effect over SAI and LAI precludes any further discussion regarding brain lateralization.

### 4.2. Hand–hand and face–hand LAI in control participants and TBPI patients

Hand sensorimotor integration can also be assessed through LAI at ISIs longer than 100 ms (Chen et al., 1999; Kotb et al., 2005; Voller et al., 2006; Turco et al., 2018a). Similar to our results for face–hand SAI, hand–hand LAI exhibited similar patterns across the four tested ISIs in the control and TBPI groups. Average normalized MEP amplitudes were below 100% in the control group at 100, 200, and 300 ms and at 100 and 200 ms in the TBPI group. The significant difference between the two groups suggests that the TBPI group had significantly higher overall amplitudes than the control group. The 95% confidence intervals in the TBPI group include zero for the 100 and 200 ms ISI but are slightly above zero for the two longer ISIs, suggesting that there might even be a tendency toward facilitation for the TBPI group.

The absence of hand–hand LAI in TBPI patients is in line with previous studies indicating that the connectivity between sensorimotor cortices and higher order areas is compromised in TBPI patients with complete injury (Qiu et al., 2014; Lu et al., 2016; Bhat et al., 2017; Rangel et al., 2021) and that cortical representation changes in TBPI are bilateral (Hsieh et al., 2002; Yoshikawa et al., 2012; Hua et al., 2013; Liu et al., 2013; Qiu et al., 2014; Fraiman et al., 2016; Kakinoki et al., 2017). It also corroborates a previous result showing a reduction in hand LAI in patients with complex regional pain syndrome, a neuropathic pain disorder (Morgante et al., 2017).

This paper is the first ever to investigate face–hand LAI. We found no evidence of face–hand LAI in either control group or TBPI patients. The absence of any inhibition in this condition might be evidence that the mechanisms underlying late sensorimotor interactions between the face and hand differ from those operating at earlier delays, and from those operating within the hand and the face (Chen et al., 1999; Kotb et al., 2005; Voller et al., 2006; Pilurzi et al., 2020).

Although we observed no face–hand inhibition for ISIs > 100ms in either group, MEP amplitudes tended toward facilitation in the TBPI group. There is some evidence that electrical stimulation of the median nerve can facilitate responses in FDI and APB at intervals from 25 to 80 ms (Deletis et al., 1992; Devanne et al., 2009; Deveci et al., 2020). The tendency toward facilitation in TBPI patients might reflect the presence of afferent-facilitation circuits between the face and the hand, although the absence of any facilitation in the control group goes against this idea. Alternatively, the facilitation might reflect broad cortical disinhibition beyond the sensorimotor cortex, which has been previously described in mammals after a nerve injury (Garraghty et al., 1991) and in humans after experimental acute deafferentation (Ziemann et al., 1998; Levy et al., 2002; Werhahn et al., 2002). Levy et al. (2002) showed decreased levels of GABA in the hemisphere contralateral to an acute deafferentation and Werhahn et al. (2002) showed increased motor cortex excitability in the hemisphere ipsilateral to an acutely deafferented upper limb related to decreased GABA-mediated cortical inhibition. The tendency toward disinhibition for the face–hand LAI condition in TBPI group suggests that a face–hand circuit involving bilateral cortical regions could be affected by reduced GABA following peripheral nerve injury.

Both SAI and LAI are modulated by GAB_A_A receptor activity (Turco et al., 2018b). Moreover, both SAI and LAI can inhibit long-interval intracortical inhibition (LICI) mediating neurons, which in turn inhibits a population of excitatory interneurons that project onto corticospinal neurons that produce the output to the spinal motoneurons (Ni et al., 2011). As a consequence, SAI may contribute to LAI, given that both will inhibit excitatory interneurons through LICI-mediating neurons. Therefore, in a context where SAI is less apparent, LAI can also be less evident, which could ultimately explain the disinhibition effects we observed for hand–hand and face–hand LAI. Interactions between cortical neural circuits may also help to explain altered AI in the uninjured limb. For instance, both short-interval intracortical inhibition (SICI) and LICI-mediating neurons inhibit short- and long-interval interhemispheric inhibition (SIHI and LIHI), affecting the output of the brain hemisphere ipsilateral to the injury (Ni et al., 2011). Accordingly, bilateral changes in sensorimotor representations (Fraiman et al., 2016; Rangel et al., 2021) and noticeable somatosensory and kinematics alterations have been described after a TBPI for the uninjured upper limb (Ramalho et al., 2019; Lustosa et al., 2022).

## 5. Limitations

Considering that injured and uninjured sides are both affected by TBPI outcomes and taking into consideration that a larger sample size could enhance statistical power, we decided to assemble all TBPI patients in a single group, although we do acknowledge the reduced number of participants as a limitation of our study. Our research group has a special interest in exploring TBPI bilateral changes in the sensorimotor cortex (Fraiman et al., 2016; Rangel et al., 2021). The ideal setting would be to consider both sides for all patients. However, that would excessively lengthen the experiment duration. Returning for a second experimental session was logistically prohibitive as most patients lived very far from the laboratory. Therefore, we made the decision to assess TBPI patients with at least partial hand function on their injured limb and patients without any hand function on their uninjured limb.

Most hand SAI studies have reported the occurrence of inhibition at intervals that correspond to the arrival of the afferent stimulus at the S1 (Tokimura et al., 2000; Bikmullina et al., 2009; Asmussen et al., 2013). As TBPI patients may have altered nerve conduction velocity due to the injured pathway (Ferrante and Wilbourn, 2002), a delay in the arrival of afferent information at S1 could be expected, hence altering the AI effect. In this sense, a useful approach would be to pair the peripheral electrical stimulation with EEG recordings, so that the ISI choice could be based on the latency of the N20 component of the somatosensory evoked potential (Di Lazzaro et al., 2002, 2005; Alle et al., 2009; Ferreri et al., 2012; Cash et al., 2015). Unfortunately, this option was not available in our context.

## 6. Conclusion

This study is the first to investigate how TBPI affects inhibitory interactions between the somatosensory and motor systems within the hand and between the face and the hand. SAI in the hand was observed for TBPI patients, a sign that the peripheral injury did not completely prevent hand sensorimotor integration. Hand LAI, however, was absent for TBPI participants, suggesting that bilateral cortical regions involved in sensorimotor integration are likely affected by the injury. As SAI contributes to LAI through LICI, at least part of the disinhibition observed for LAI could be explained by the SAI inhibitory shift in ISIs induced by TBPI.

Our results point to a bilateral central reorganization involving hand and face sensorimotor representations. This was noted through the different face–hand SAI patterns observed for the control group and the TBPI patients. For long-latency intervals, there was a tendency toward facilitation in the TBPI group. These outcomes suggest that hand–hand and face–hand sensorimotor circuits are affected by plastic changes after an upper limb nerve injury, corroborating the existence of an inhibitory regulation system between the representations of the face and the hand that seems to be altered in TBPI. Remarkably, these findings also reinforce the idea that changes arising from TBPI are not restricted to the injured limb and that cortical alterations resulting from a unilateral peripheral injury can extend to both hemispheres.

## Data availability statement

The raw data supporting the conclusions of this article will be made available by the authors, without undue reservation.

## Ethics statement

The studies involving human participants were reviewed and approved by Comitê de Ética em Pesquisa Instituto de Neurologia Deolindo Couto–Universidade Federal do Rio de Janeiro. The patients/participants provided their written informed consent to participate in this study. Written informed consent was obtained from the individual(s) for the publication of any potentially identifiable images or data included in this article.

## Author contributions

FT, BR, KR, and CV conceived and planned the experiments. FT, MR, AS, and VM carried out the experiments and contributed to data processing. FT performed data analysis and took the lead in writing the manuscript. FT, BR, RC, and CV contributed to the interpretation of the results. All authors provided critical feedback and helped shape the research, analysis, and manuscript.
