# Supplementary material for: Plasticity of face–hand sensorimotor circuits after a traumatic brachial plexus injury
Source: Front Neurosci. 2023 Aug 7;17:1221777. doi: 10.3389/fnins.2023.1221777 (PMC10440702; doi:10.3389/fnins.2023.1221777)
Supplement: Supplementary file 1 [file Table_1.DOCX]

**Table S.1** Results are mean ± standard deviations. Between-group comparisons were performed using unpaired T tests with Welch's correction. TBPI-I, traumatic brachial plexus injury participants assessed on the injured side; TBPI-UI, traumatic brachial plexus injury participants assessed on the uninjured side; TMS-only MEP amplitude, mean motor evoked potential amplitude when transcranial magnetic stimulation was applied without previous peripheral electrical stimulation; 1mV/MT, motor threshold to elicit 1 mV responses, MSO, maximum stimulator output; pET, peripheral electrical threshold for the finger and the face; pESI, peripheral electrical stimulation intensity used during AI experiments set at 3xpET for the finger and 2xpET for the face.

| **Transcranial Magnetic Stimulation and Peripheral Electrical Stimulation Parameters** | | |
| --- | --- | --- |
|  | TBPI-I Subgroup (n=5) | TBPI-UI Subgroup (n=4) |
| TMS-only MEP amplitude (mV) | 0.73 ± 0.34 | 0.98 ± 0.47 |
| 1 mV/MT (% MSO) | 50.60 ± 9.02 | 54.50 ± 6.86 |
| Finger pET (mA) | 4.62 ± 1.75 | 3.90 ± 0.55 |
| Face pET (mA) | 2.96 ± 2.08 | 1.48 ± 0.51 |
| Finger pESI (mA) | 13.86 ± 5.25 | 10.28 ± 1.84 |
| Face pESI (mA) | 5.92 ± 4.16 | 2.85 ± 1.09 |
