# Supplementary material for: Plasticity of face–hand sensorimotor circuits after a traumatic brachial plexus injury
Source: Front Neurosci. 2023 Aug 7;17:1221777. doi: 10.3389/fnins.2023.1221777 (PMC10440702; doi:10.3389/fnins.2023.1221777)
Supplement: Supplementary file 2 [file Data_Sheet_1.PDF]

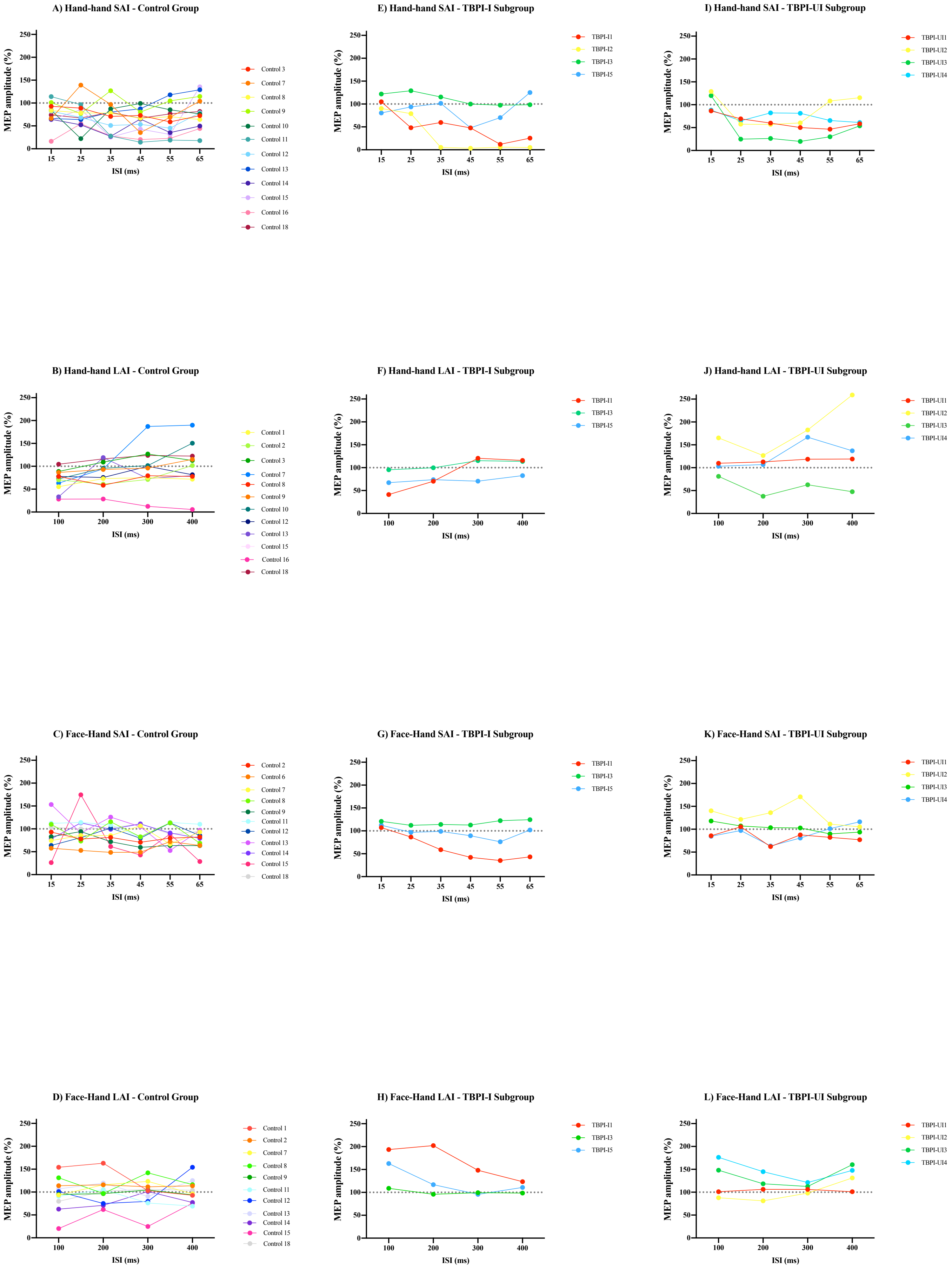
A) Hand-hand SAI - Control Group
MEP amplitude (%)
ISI (ms)
Control 3
Control 7
Control 8
Control 9
Control 10
Control 11
Control 12
Control 13
Control 14
Control 15
Control 16
Control 18
E) Hand-hand SAI - TBPI-I Subgroup
TBPI-I1
TBPI-I2
TBPI-I3
TBPI-I5
I) Hand-hand SAI - TBPI-UI Subgroup
TBPI-UI1
TBPI-UI2
TBPI-UI3
TBPI-UI4
B) Hand-hand LAI - Control Group
Control 1
Control 2
Control 3
Control 7
Control 8
Control 9
Control 10
Control 12
Control 13
Control 15
Control 16
Control 18
F) Hand-hand LAI - TBPI-I Subgroup
TBPI-I1
TBPI-I3
TBPI-I5
J) Hand-hand LAI - TBPI-UI Subgroup
TBPI-UI1
TBPI-UI2
TBPI-UI3
TBPI-UI4
C) Face-Hand SAI - Control Group
Control 2
Control 6
Control 7
Control 8
Control 9
Control 11
Control 12
Control 13
Control 14
Control 15
Control 18
G) Face-Hand SAI - TBPI-I Subgroup
TBPI-I1
TBPI-I3
TBPI-I5
K) Face-Hand SAI - TBPI-UI Subgroup
TBPI-UI1
TBPI-UI2
TBPI-UI3
TBPI-UI4
D) Face-Hand LAI - Control Group
Control 1
Control 2
Control 7
Control 8
Control 9
Control 11
Control 12
Control 13
Control 14
Control 15
Control 18
H) Face-Hand LAI - TBPI-I Subgroup
TBPI-I1
TBPI-I3
TBPI-I5
L) Face-Hand LAI - TBPI-UI Subgroup
TBPI-UI1
TBPI-UI2
TBPI-UI3
TBPI-UI4
