# Supplementary material for: Plasticity of face–hand sensorimotor circuits after a traumatic brachial plexus injury
Source: Front Neurosci. 2023 Aug 7;17:1221777. doi: 10.3389/fnins.2023.1221777 (PMC10440702; doi:10.3389/fnins.2023.1221777)
Supplement: Supplementary file 3 [file Data_Sheet_2.docx]

**Figure S.1** Example of participant positioning during the experimental session, front view (A), side view (B) and detailed view (C). Participants were seated in a comfortable chair with an arm support that maintained the shoulder at a neutral position, elbow flexed at approximately 90°, forearm and wrist in a neutral position and hand relaxed.

**Figure S.2** Short Afferent Inhibition (SAI) and Long Afferent Inhibition (LAI) results. A, C- Hand-hand interaction: peripheral electrical stimulation applied on the tip of the index finger followed by contralateral transcranial magnetic stimulation over the first dorsal interosseous hot spot. B, D- Face-hand interaction: Peripheral electrical stimulation applied on the face, above the upper lip, followed by contralateral transcranial magnetic stimulation over the first dorsal interosseous hot spot. Different interstimulus intervals were applied (15-65 ms for SAI and 100-400 ms for LAI). Group mean motor evoked potential amplitudes for each interstimulus interval was normalized to the TMS-only mean MEP amplitude (transcranial magnetic stimulation without previous peripheral electrical stimulation) in that experimental condition. Control Group (blue); TBPI-I subgroup, TBPI patients assessed on the injured side (green), and TBPI-UI subgroup, TBPI patients assessed on the uninjured side (red). Bars represent the standard error of the mean. Values below the dotted line at 100% indicate an inhibition effect, values above the dotted line indicate a disinhibition effect. Repeated measures one-way ANOVA and Dunnett’s post-test were performed for within group analysis (CG, TBPI-I and TBPI-UI subgroups). Two-way ANOVAs and Tukey’s multiple comparison test were used for between-group comparisons (CG, TBPI-I and TBPI-UI subgroups). * = p < 0.05; * above bar indicates significant result at within group analysis and * above brackets indicate significant results at between-group analysis. None of the ANOVAs revealed an ISI x Group interaction (hand-hand SAI: F_12, 119_ = 0.7785, p=0.672; face-hand SAI: F_12, 105_ = 0.4753, p=0.925; hand-hand LAI: F_8, 75_ = 0.4641, p=0.878; face-hand LAI: F_8, 75_ = 1.229, p=0.294). There was no main effect of Group for hand-hand SAI (F_2, 119_ = 0.03919, p=0.962), or face-hand SAI (F_2, 105_ = 2.698, p=0.072). But for hand-hand LAI (F_2, 75_ = 4.230, p=0.018) and face-hand LAI (F_2, 75_ = 6.381, p=0.0028) there was a main effect of Group.

**Figure S.3** Results presented by groups with individual mean normalized values. Left column displays results for the Control Group for Hand-hand SAI (A), Hand-hand LAI (B), Face-hand SAI (C) and Face-hand LAI (D). Center column displays results for the TBPI-I subgroup (traumatic brachial plexus injury patients assessed on the injured side) for Hand-hand SAI (E), Hand-hand LAI (F), Face-hand SAI (G) and Face-hand LAI (H). Right column displays results for the TBPI-UI subgroup (traumatic brachial plexus injury patients assessed on the uninjured side) for Hand-hand SAI (I), Hand-hand LAI (J), Face-hand SAI (K) and Face-hand LAI (L). Values below the dotted line at 100% indicate an inhibition effect, values above the dotted line indicate a disinhibition effect.
