# Supplementary figures and images for: Plasticity of face–hand sensorimotor circuits after a traumatic brachial plexus injury
Source: Front Neurosci. 2023 Aug 7;17:1221777. doi: 10.3389/fnins.2023.1221777 (PMC10440702; doi:10.3389/fnins.2023.1221777)

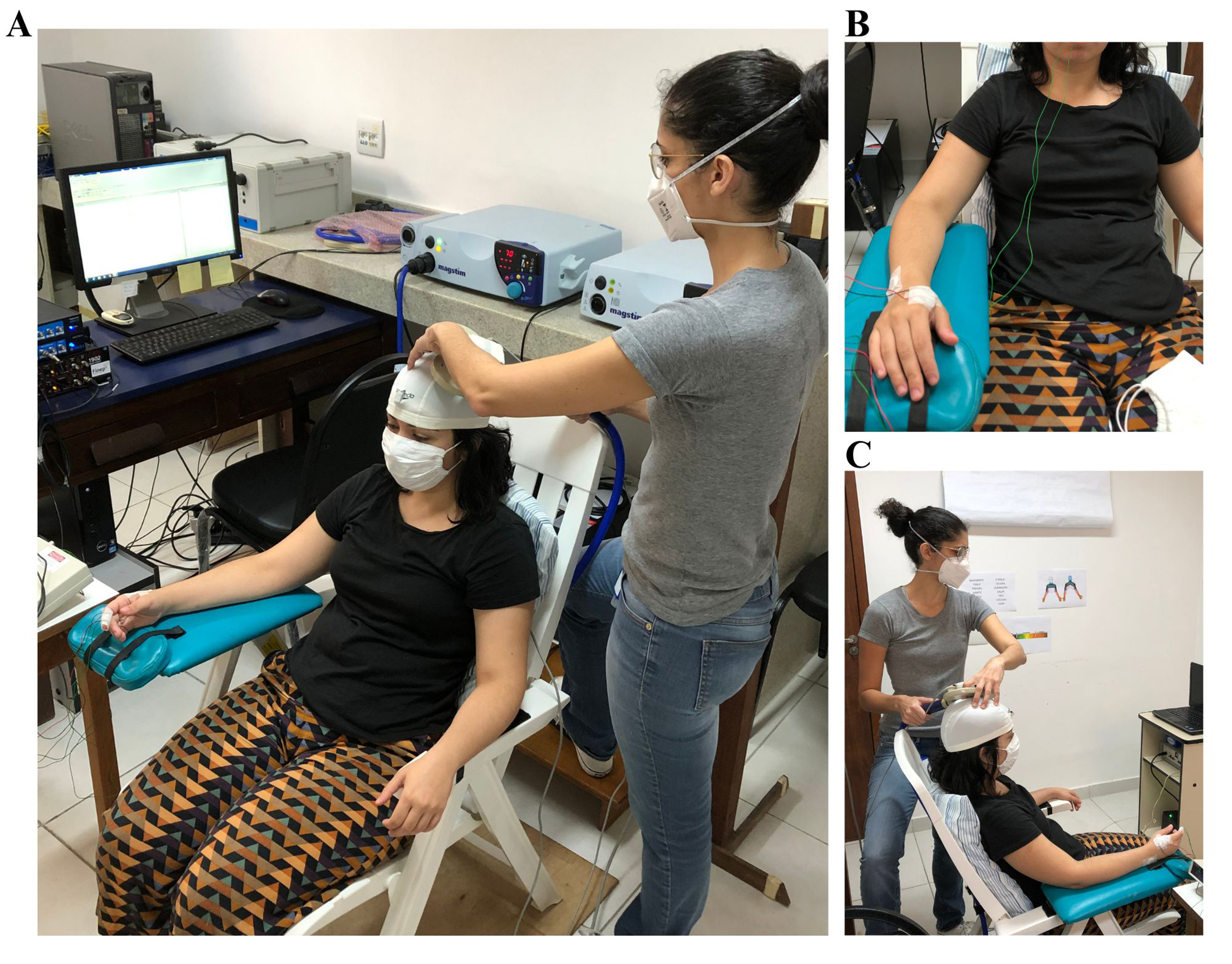

Supplement: Supplementary file 4 [file Image_1.JPEG]

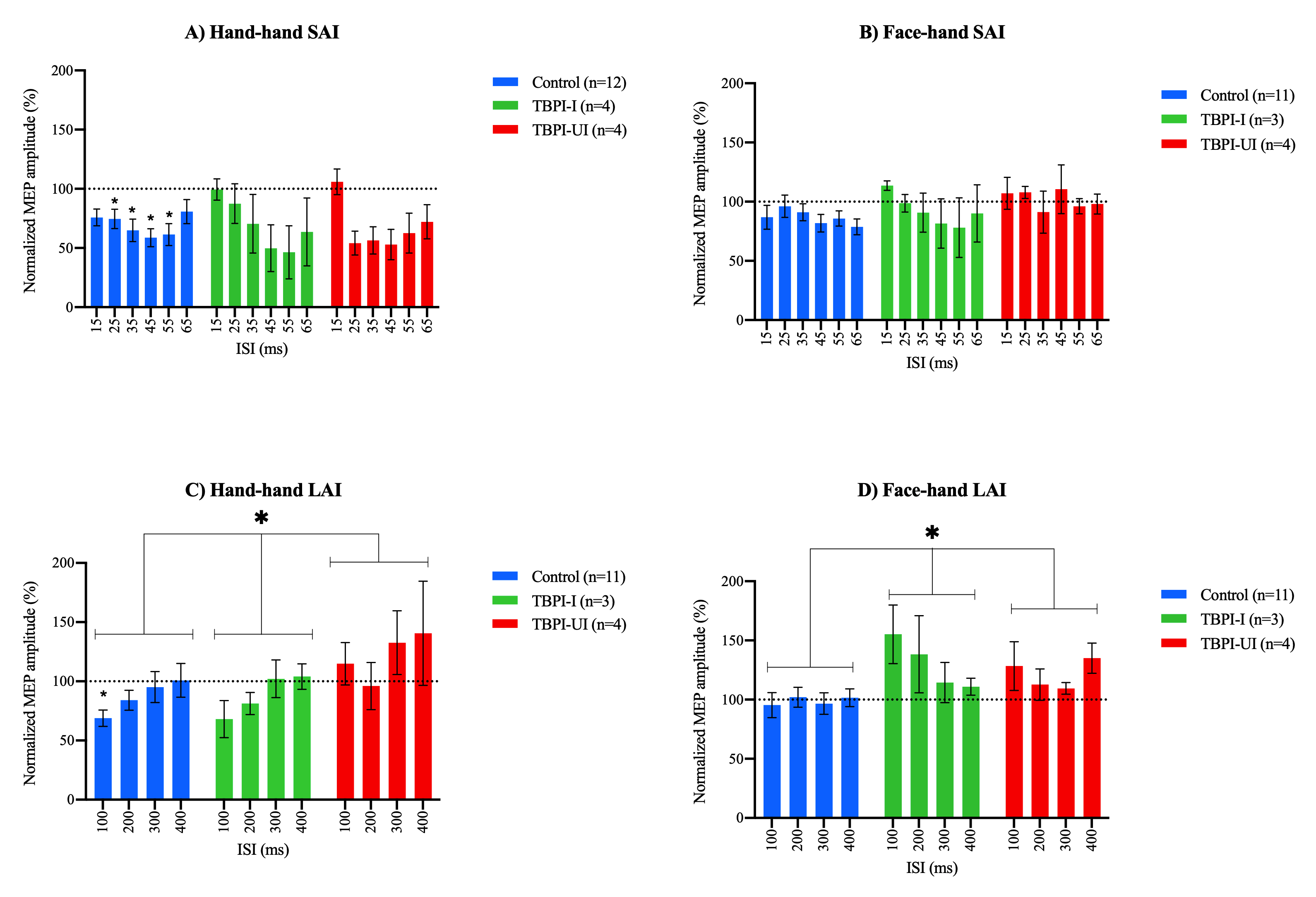

Supplement: Supplementary file 5 [file Image_2.TIFF]
